# A Case of Severe Injury of the Orbit

**Published:** 1882-11

**Authors:** E. L. Holmes, Roswell Park

**Affiliations:** Attending Surgeon; Assistant Surgeon to the Illinois Charitable Eye and Ear Infirmary


### Selections.

A CASE OF SEVERE INJURY OF THE ORBIT. By E. L. HOLMES, M.D., Attending Surgeon, and ROSWELL PARK, M.D., Assistant Surgeon to the Illinois Charitable Eye and Ear Infirmary.*

O. J., a boy fourteen years of age, was brought from Wisconsin to the dispensary of the infirmary, June 13, 1881, with the following history of an accident which happened eight months before.

A piece of pine wood, thrown with great force from a rapidly revolving circular saw, penetrated the left orbit, through the middle of the lower lid. The local surgeon succeeded in removing only a few splinters.

An examination revealed a small fistulous opening, surrounded with polypoid granulations, in the integument of the lower lid; also a similar growth at the lower portion of the cornea, with considerable redness and œdema of the lower part of the ocular conjunctiva. The contracted pupil and cloudiness of the cornea prevented a precise diagnosis of intra-ocular changes. There were slightly diminished tension of the globe, great loss of vision, with some tenderness and swelling of the side of the face, and impediment in the motion of the jaw.

It was difficult to determine, by probing, the direction in which the piece entered the orbit or from which the discharge came.

After the administration of ether, an incision was made along the border of the orbit, through the integument at the fistulous opening. The finger could then detect a piece of wood, firmly surrounded at its lower end by hard tissue. This piece extended upward between the conjunctiva and sclerotic, to the lower border

* Reprinted from the *Archives of Ophthalmology*, Vol xi, No. 1, March, 1882.

of the cornea. A careful examination seemed to demonstrate that the other portion of the stick extended into the antrum. And yet there was no history of discharge of blood or pus from the nostril. The free end, lying between the conjunctiva and sclerotic, was easily removed through the incision in the lid, but broke off close to the bone.

A careful examination with the tip of the finger disclosed a small piece of wood corresponding to the piece broken off, but held firmly by the products of inflammation. It seemed to extend directly downward. As an operation involving other tissues than those of the orbit appeared necessary, the case was placed under the charge of Dr. R. Park. No theory seems satisfactorily to explain how the piece behind the conjunctiva was at a right angle to the "stick" finally removed by Dr. Park.

SUBSEQUENT ACCOUNT BY DR. R. PARK.

It having been the unanimous opinion of all who first saw the case that a comparatively small sliver of wood probably extended into the antrum, I proceeded to try to effect its removal. Accordingly, the following day, having anæsthetized the boy again, I prolonged the incision made by Dr. Holmes toward the inner canthus, and then down the side of the nose. Reflecting the flap thus made, I perforated and entered the antrum. Prolonged exploration with the finger and probe failed to reveal the splinter. I again explored the orbit, and could grasp the piece with the forceps, but not tightly enough to remove it, nor could I convince myself as to its exact position, since the swelling from inflammation and the previous operation interfered materially with any search.

In the meantime over an hour had elapsed; the parts being very vascular, the boy being weakened by free hæmorrhage and two consecutive operations, and, moreover, beginning to tolerate the anæsthetic badly, it was decided to postpone further operative measures. Accordingly, a drainage tube was inserted into the antrum; the incisions were closed with silk sutures, and covered with dressings of cold water and alcohol.

The incisions healed well, although there was copious discharge from the tube. The boy's mother being anxious to take him home to avoid expense, she was allowed to leave with him one week after the operation, with directions as to his care, and especially as to keeping me informed as to his condition.

Twice during September I heard that there was still discharge from the drainage tube, which had been allowed to remain.

Finally, after some further correspondence, he came to the city in November, or about five months after his first visit.

*Status præsens.*—The tube having been removed a month before, the lines of my incision are all healed, and with surprisingly little cicatrix. There are more or less swelling and induration of all the tissues of the temporo-facial region. The mouth can only be opened to extent of three-fourths of an inch; there is more tenderness over the left temporo-maxillary articulation than anywhere else. There is a sinus at the site of the original injury, through which a curved probe passes downward and backward more than an inch without touching diseased bone. There is a fistulous opening on the left side of the soft palate, close to the hard palate. There is discharge from both these openings. According to the mother, there has at several times during the summer, been a discharge from the left ear. There is now no visible opening or perforation of the membrana tympani through which it could have come. H. D., left ear=$\frac{15}{30}$; right ear,=$\frac{30}{30}$. Membrana tympani is slightly thickened; light spot irregular. There is coloboma above from simple atrophy of iris structure. This condition gives the pupil an oval slit shape. Media cloudy; details of fundus invisible. The patient counts fingers at six inches if held above the level of the eye. The globe is almost immovable in the orbit, held so by the products of inflammation.

Nov. 17, 1881.—With the assistance of Drs. Curtis, Tilley, and others, the boy being under the influence of chloroform (with one per cent. of amyl nitrite) I proceeded as follows: I first plugged the left posterior naris, and then laid up a flap of the cheek by a curved incision from the outer angle of the orbit to the angle of the mouth. Coming thus upon the site of the operation five months previous, I found the antrum pretty well filled up with granulation tissue. A probe could now be passed downward and backward and through the fistula in the soft palate. Finding nothing in the antrum, I then removed, with chisel, saw and forceps, the inner half of the malar bone and almost all the floor af the orbit; taking care, as far as possible, to peel off the periosteum before attacking the bone. Probing and searching toward the nasal side of the orbit, I found nothing suspicious, and had begun to despair of finding anything to reward my efforts. I resolved, however, to work outward, and removing more of the malar bone and outer wall and floor of the orbit, I at last touched with the probe something which felt very much like wood. Clearing away the bone a little more, I caught with strong forceps and with some force extracted the foreign body. This was a piece of pine two and one-half inches long, and one-half inch square at its base. It had been lying with its anterior extremity engaged on a level below the infra-orbital ridge, and on the external surface of the orbit; its posterior extremity, judging from its length and now evident direction, was engaged just in front of the maxillary articulation. In its entrance and course it must, therefore, have penetrated the infero-exterior wall of the orbit; having been thus lodged with one end in the orbit, the other in the zygomatic fossa, and lying across the outer extremity of the spheno-maxillary fissure.

On further examination finding no other fragments, I curetted the fistulous tracks, and then united the edges of the flaps, after inserting a drainage tube. The progress of the wound was satisfactory in every particular.

On December 22, the patient returned to his home. His condition at that time was as follows: Line of incision entirely healed. No change in condition of parts within the globe. The eye is now freely movable, and its movements follow those of the other eye quite accurately, except upward. Its upward motion is interfered with by a condition of ectropion, combined with a band of granulation tissue, forming a species of symblepharon. The ectropion is caused by the cicatrix following the injury and the operations. At some time in the future a plastic operation may be made for its partial relief; it was thought best to postpone this for a few months. Region supplied by the infra-orbital nerve entirely anæsthetic. V=fingers at five ft. in good light. Hearing distance, left ear, $\frac{25}{30}$.

